# Hay Fever, or Rhinitis Vasco-motor Periodica, and Its Radical Cure

**Published:** 1888-11

**Authors:** 


HAY FEVER, OR RHINITIS VASCO-MOTOR PERIODICA, AND ITS RADICAL CURE. By E. Lippincott, M. D. Chicago: Gross & Delbridge. 1888.

There is scarcely a disease on the nosological list upon which physicians desire more light than on the so called hay fever. One, therefore, turns with hopeful expectation to Dr. Lippincott's little volume. The Doctor considers the cause of this disease to be due to a local chronic affection which is periodically aggravated by inhaled irritants. For its treatment he praises Naphthalin very highly; other remedies are mentioned, but the indications given for their use are very meagre. The theory of the psoric origin of this disease Dr. Lippincott does not credit, nor does he state the *primary cause* of the chronic local disease to which he attributes the origin of this disease.

